# Precision Targeting of *KRAS*-Mutant Cancers: Beyond G12C Toward G12D and Pan-RAS Therapeutic Strategies

**DOI:** 10.3390/ijms27135796

**Published:** 2026-06-26

**Authors:** Yoshihito Kano

**Affiliations:** Department of Medical Oncology, Graduate School of Medical and Dental Sciences, Institute of Science Tokyo, 1-5-45 Yushima, Bunkyo-Ku, Tokyo 113-8510, Japan; kano.canc@md.isct.ac.jp

**Keywords:** KRAS G12C, KRAS G12D, RAS(ON) inhibitors, pan-RAS targeting, liquid biopsy, targeted protein degradation, precision oncology

## Abstract

*KRAS* is one of the most frequently mutated oncogenes in human cancer and has long been considered an “undruggable” therapeutic target because of its high affinity for guanine nucleotides and limited druggable binding pockets. Recent advances in structural biology and molecular pharmacology have transformed this paradigm, leading to the successful development of KRAS G12C inhibitors such as sotorasib and adagrasib. These agents established proof-of-concept for direct KRAS inhibition and marked an important advance in precision oncology. However, intrinsic and acquired resistance mechanisms, adaptive signaling reactivation, and tumor heterogeneity continue to limit the durability of clinical responses. Therapeutic development has rapidly expanded beyond KRAS G12C toward broader strategies including KRAS G12D inhibitors, pan-RAS and RAS(ON) inhibitors, degraders, and biomarker-guided combination approaches. In parallel, circulating tumor DNA (ctDNA) and other biomarker-driven strategies are increasingly enabling dynamic monitoring of treatment response, minimal residual disease, and resistance evolution. In this review, we summarize the molecular biology and conformational regulation of KRAS signaling, recent advances in allele-specific and pan-RAS therapeutic strategies, mechanisms of resistance, and emerging precision oncology frameworks for *KRAS*-mutant cancers.

## 1. Introduction

*KRAS* is one of the most frequently mutated oncogenes in human cancer and serves as a major driver of tumor initiation, progression, and therapeutic resistance. Activating *KRAS* mutations are particularly prevalent in pancreatic ductal adenocarcinoma (PDAC), colorectal cancer (CRC), and non-small-cell lung cancer (NSCLC), where they are associated with aggressive tumor biology and poor clinical outcomes [1,2,3].

KRAS is a small guanosine triphosphatase (GTPase) that regulates multiple signaling pathways involved in cell proliferation, survival, metabolism, and differentiation. Under physiologic conditions, KRAS cycles between inactive GDP-bound and active GTP-bound states through interactions with guanine nucleotide exchange factors (GEFs) and GTPase-activating proteins (GAPs) [4]. Oncogenic mutations impair GTP hydrolysis and promote constitutive activation of downstream signaling pathways, most notably the RAF–MEK–ERK and PI3K–AKT cascades.

Despite its biological importance, KRAS was long considered an “undruggable” target because of its high affinity for GTP/GDP and the lack of obvious druggable binding pockets [2,3]. This paradigm changed following the discovery of the switch-II pocket, which enabled development of covalent KRAS G12C inhibitors such as sotorasib and adagrasib [5,6,7]. These agents established proof-of-concept for direct KRAS inhibition and represented a major proof-of-concept in precision oncology. However, clinical efficacy remains limited by intrinsic and acquired resistance, adaptive pathway reactivation, and tumor heterogeneity.

Recent studies have further demonstrated that KRAS signaling is dynamically regulated through mutation-specific conformational changes and post-translational modifications. Src-mediated phosphorylation of Tyr32 and Tyr64 alters switch I and II conformations and modulates KRAS signaling through SHP2-dependent regulation [8,9]. In addition, distinct *KRAS* alleles exhibit unique biochemical and structural properties that influence therapeutic sensitivity and adaptive signaling behavior, underscoring the importance of mutation-specific KRAS biology.

Therapeutic development has therefore rapidly expanded beyond KRAS G12C toward KRAS G12D inhibitors, pan-RAS and RAS(ON) inhibitors, and rational combination strategies involving EGFR, SHP2, SOS1, MEK, ERK, immunotherapy, and chemotherapy. Furthermore, circulating tumor DNA (ctDNA) and other biomarker-driven approaches are increasingly enabling dynamic monitoring of treatment response, minimal residual disease, and resistance evolution.

Several authoritative reviews have summarized RAS biology, the historical development of KRAS G12C inhibitors, and emerging therapeutic strategies for RAS-mutant cancers [10,11]. However, the field has rapidly moved beyond the initial G12C paradigm, creating a need for an integrated framework that connects non-G12C allele biology, nucleotide-state selectivity, tumor-lineage constraints, resistance evolution, and biomarker-guided treatment.

The distinctive contribution of this review is to organize KRAS-targeted therapy through a post-G12C precision oncology framework. Specifically, we compare OFF-state trapping, non-covalent allele-specific inhibition, RAS(ON)/pan-RAS targeting, and degradation-based strategies; evaluate how these approaches differ across tumor contexts such as NSCLC, CRC, and PDAC; and discuss how ctDNA and longitudinal molecular profiling may support adaptive treatment strategies. Thus, this review is intended not as a comprehensive catalog of KRAS-targeted agents, but as a conceptual synthesis of how therapeutic logic is shifting from single-allele inhibition toward state-selective, lineage-aware, and biomarker-guided KRAS precision oncology.

In this context, we summarize the molecular biology of KRAS signaling, recent advances in allele-specific and pan-RAS therapeutic strategies, mechanisms of resistance, and emerging precision oncology approaches for *KRAS*-mutant cancers.

This review is structured around two central questions. First, how do non-G12C, RAS(ON), and pan-RAS strategies change the therapeutic logic established by KRAS G12C inhibition? Second, which biological and clinical variables—including *KRAS* allele, nucleotide/conformational state, tumor lineage, resistance mechanism, and biomarker dynamics—should guide the choice between allele-specific and broader RAS-targeted therapeutic strategies? Sections on KRAS biology, undruggability, and G12C inhibition are therefore presented as focused background for understanding the post-G12C therapeutic framework rather than as a chronological account of the entire KRAS field.

## 2. Review Strategy and Evidence Classification

This article is a narrative review with a scoping approach and was not designed as a systematic review or meta-analysis. Literature was identified through searches of PubMed, ClinicalTrials.gov, regulatory documents, major oncology conference abstracts, and publicly available company disclosures. Searches were focused on studies published or publicly available up to May 2026. Key search terms included “KRAS,” “KRAS G12C,” “KRAS G12D,” “pan-RAS,” “RAS(ON),” “SHP2 inhibitor,” “SOS1 inhibitor,” “KRAS degrader,” “KRAS resistance,” “ctDNA,” and “KRAS-mutant cancer.”

Priority was given to peer-reviewed original studies, randomized clinical trials, regulatory documents, and clinical trial registry records. Conference abstracts and company disclosures were included only when peer-reviewed data were unavailable for rapidly evolving agents or late-breaking clinical trial results; in such cases, the preliminary or non-peer-reviewed nature of the evidence is indicated in the text or tables. Preclinical studies were included when they provided mechanistic insight or proof-of-concept for emerging therapeutic strategies.

To avoid overinterpretation, evidence was categorized according to maturity: Level 1, approved therapy or randomized phase III evidence; Level 2, peer-reviewed phase I/II clinical evidence; Level 3, clinical trial registry data, conference abstracts, or company disclosures without mature peer-reviewed publication; Level 4, preclinical evidence; and Level 5, conceptual or hypothesis-generating strategies. Conclusions were calibrated according to this evidence hierarchy.

## 3. KRAS Biology and Oncogenic Signaling

### 3.1. Structure and Function of KRAS

KRAS is a member of the RAS family of small guanosine triphosphatases (GTPases) that regulate cell proliferation, survival, metabolism, and differentiation. Among the major RAS isoforms, *KRAS* is the most frequently mutated in human cancers. Structurally, KRAS consists of a conserved G-domain responsible for nucleotide binding and hydrolysis, and a C-terminal hypervariable region involved in membrane localization [4]. The G-domain contains several critical regions, including the phosphate-binding loop (P-loop), switch I, and switch II regions, which regulate nucleotide binding, effector interaction, and conformational dynamics [5]. In particular, the switch regions undergo conformational changes depending on GDP- or GTP-bound status and play central roles in KRAS signaling and therapeutic vulnerability.

Under physiologic conditions, KRAS cycles between inactive GDP-bound and active GTP-bound states through regulation by guanine nucleotide exchange factors (GEFs) and GTPase-activating proteins (GAPs). Activated KRAS interacts with downstream effectors including RAF, PI3K, and RalGDS, thereby promoting signaling pathways involved in cellular growth and survival. Oncogenic *KRAS* mutations impair intrinsic or GAP-mediated GTP hydrolysis, resulting in persistent activation of downstream signaling. Importantly, recent studies indicate that KRAS is not a static binary switch but rather a highly dynamic protein capable of adopting multiple conformational states. These conformational properties have become central to the development of modern KRAS-targeted therapeutics [2,6,7].

### 3.2. KRAS GDP/GTP Cycling and Downstream Signaling

KRAS activity is regulated through tightly coordinated GDP/GTP cycling. GEFs such as SOS1 promote GDP release and GTP loading, whereas GAPs including NF1 accelerate GTP hydrolysis and terminate signaling [4]. In the active GTP-bound state, conformational rearrangements within the switch I and II regions facilitate interaction with downstream effector proteins.

The RAF–MEK–ERK pathway represents the canonical downstream signaling cascade activated by KRAS and plays a major role in tumorigenesis. KRAS also activates the PI3K–AKT–mTOR and RalGDS pathways, which contribute to cell survival, metabolism, cytoskeletal remodeling, and metastatic behavior. Oncogenic *KRAS* mutations disrupt normal GDP/GTP cycling and promote constitutive downstream signaling independent of upstream receptor tyrosine kinase activation. Importantly, KRAS signaling is further modulated by conformational regulation and post-translational modifications. Src-mediated phosphorylation of Tyr32 and Tyr64 alters switch-region dynamics and promotes formation of a signaling-impaired “dark-state” KRAS conformation, whereas SHP2-mediated dephosphorylation restores canonical signaling activity [8]. These findings highlight the complex interplay between nucleotide cycling, conformational regulation, and therapeutic vulnerability.

### 3.3. Biological Differences Among KRAS Mutations

Although *KRAS* mutations are often collectively classified as a single oncogenic entity, distinct *KRAS* alleles exhibit substantial biochemical and functional heterogeneity [1,4,12]. The majority of *KRAS* mutations occur at codons 12, 13, and 61, with common variants including G12D, G12V, G12C, G13D, and Q61H [3]. Codon 12 and 13 mutations primarily impair GAP-mediated GTP hydrolysis, whereas codon 61 mutations directly disrupt intrinsic GTP hydrolysis and often produce stronger constitutive activation. These mutation-specific differences influence signaling dependency, therapeutic sensitivity, and adaptive resistance [13]. KRAS G12C has emerged as the most therapeutically tractable allele because the mutant cysteine residue enables covalent inhibitor binding within the switch-II pocket [5,6,7]. Importantly, KRAS G12C retains substantial nucleotide cycling capacity, allowing inhibitors to selectively target the inactive GDP-bound state. Unlike KRAS G12C, KRAS G12D lacks a targetable cysteine residue for covalent inhibition [12,14,15]. KRAS G12D-driven tumors, particularly PDAC, display strong MAPK dependency and extensive tumor microenvironment remodeling, supporting the development of non-covalent allele-specific inhibitors and rational combination strategies. Codon 61 mutations such as Q61H represent another biologically distinct subgroup characterized by profound impairment of GTP hydrolysis and relative independence from upstream SOS1/SHP2-mediated regulation [13,16]. These properties contribute to resistance against certain upstream-targeted therapeutic approaches. Collectively, these findings demonstrate that *KRAS* mutations are biologically heterogeneous and support the transition from mutation-agnostic treatment toward allele-specific precision therapeutic strategies.

## 4. Why KRAS Was Historically “Undruggable”

For decades, KRAS was considered an “undruggable” oncogenic target because of several intrinsic structural and biochemical challenges. KRAS binds GDP and GTP with exceptionally high affinity, making competitive inhibition of nucleotide binding extremely difficult. In addition, KRAS possesses a relatively smooth protein surface lacking deep hydrophobic pockets suitable for conventional small-molecule binding [2,3]. Another major obstacle was the difficulty of disrupting KRAS-mediated protein–protein interactions with downstream effectors such as RAF and PI3K. Early therapeutic approaches, therefore, focused largely on indirect strategies, including inhibition of KRAS membrane localization through farnesyltransferase inhibitors (FTIs) [17,18]. However, FTIs failed to demonstrate substantial clinical efficacy, in part because KRAS and NRAS can undergo alternative geranylgeranylation, allowing membrane localization and signaling to persist despite farnesyltransferase inhibition [19]. This paradigm changed following the discovery of the switch-II pocket (S-IIP), a transient binding site adjacent to the switch II region. Ostrem and colleagues demonstrated that covalent small molecules could selectively target KRAS G12C through this pocket, establishing proof-of-concept for direct KRAS inhibition [5]. This mechanistic advance highlighted the importance of KRAS conformational dynamics and paved the way for modern allele-specific and state-selective KRAS-targeted therapies.

## 5. Clinical Development of KRAS G12C Inhibition

**Figure 1:**
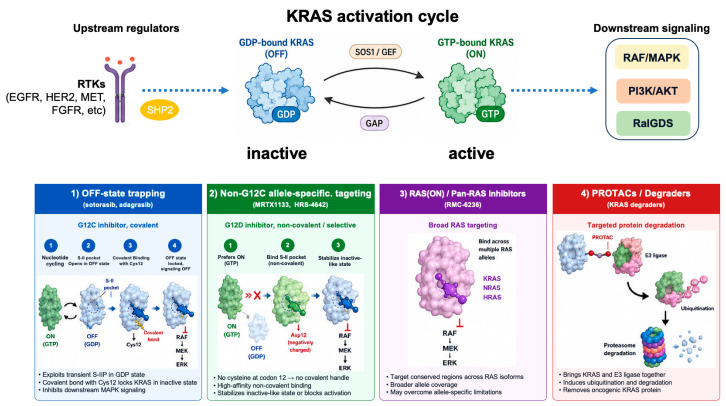
Mechanistic classes of KRAS-targeted therapies. The **Upper panels** compare key mechanistic features and illustrate the canonical KRAS GDP/GTP activation cycle. The **Lower Panels**: KRAS-targeted strategies have evolved from allele-specific KRAS G12C inhibition to KRAS G12D inhibition, multi-selective RAS targeting/RAS(ON) active-state inhibition, and targeted protein degradation. KRAS G12C inhibitors covalently bind the switch-II pocket of GDP-bound KRAS G12C and trap KRAS in an inactive OFF state, whereas KRAS G12D inhibitors require non-covalent approaches because G12D lacks a reactive cysteine residue. Pan-RAS inhibition describes the breadth of RAS allele or isoform coverage, while RAS(ON) inhibition describes state-selective targeting of active GTP-bound RAS. These concepts can overlap; multi-selective RAS(ON) inhibitors such as daraxonrasib/RMC-6236 broadly target active-state RAS proteins. Targeted degraders recruit E3 ubiquitin ligases to induce KRAS ubiquitination and proteasomal degradation.

The development of KRAS G12C inhibitors represents a major breakthrough in precision oncology (Table 1). Advances in structural biology and covalent drug design enabled direct targeting of KRAS G12C, transforming KRAS from a historically “undruggable” oncogene into a clinically actionable therapeutic target. The major mechanistic classes of KRAS-targeted therapies, including allele-specific G12C and G12D inhibitors, multi-selective RAS(ON)/pan-RAS inhibitors, allele-selective RAS(ON) inhibitors, and KRAS degraders, are summarized in Figure 1.

### 5.1. Discovery of the Switch-II Pocket

A pivotal advance in KRAS drug discovery was the identification of the switch-II pocket (S-IIP), a transient binding site adjacent to the switch II region. Ostrem and colleagues demonstrated that covalent small molecules could selectively bind the mutant cysteine residue of KRAS G12C and trap KRAS in its inactive GDP-bound state [5]. This study established proof-of-concept for direct KRAS inhibition and highlighted the importance of KRAS conformational dynamics in therapeutic targeting [6].

### 5.2. Sotorasib

Sotorasib (AMG 510) was the first KRAS G12C inhibitor approved for clinical use [20,21,22,23]. By covalently binding the switch-II pocket of GDP-bound KRAS G12C, sotorasib suppresses downstream MAPK signaling [20,21]. In the phase II NSCLC cohort of CodeBreaK 100, sotorasib achieved an objective response rate of approximately 41%, with a median progression-free survival of 6.3 months and a median overall survival of 12.5 months. These data led to accelerated FDA approval in 2021 [22,23,24]. In the randomized phase III CodeBreaK 200 trial, sotorasib significantly improved progression-free survival compared with docetaxel in previously treated KRAS G12C-mutant NSCLC (median PFS, 5.6 vs. 4.5 months; HR, 0.66). The objective response rate was also higher with sotorasib than with docetaxel (28.1% vs. 13.2%), while median overall survival was not improved with sotorasib compared with docetaxel (10.6 vs. 11.3 months; HR, 1.01) [25] (Table 2A). However, therapeutic efficacy differed across tumor types, with lower response rates observed in colorectal cancer because of rapid EGFR-mediated adaptive signaling reactivation [26,27,28].

### 5.3. Adagrasib

Adagrasib (MRTX849) is another covalent KRAS G12C inhibitor with demonstrated clinical activity in KRAS G12C-mutant cancers [29]. The KRYSTAL clinical trial program showed durable responses in heavily pretreated NSCLC patients, leading to FDA approval. An important feature of adagrasib is its central nervous system (CNS) activity, including intracranial responses in patients with active brain metastases [30,31]. Compared with sotorasib, adagrasib exhibits a longer half-life and sustained target inhibition, although the clinical significance of these pharmacologic differences remains under investigation. In KRYSTAL-1, adagrasib plus cetuximab showed an objective response rate of 34.0%, median PFS of 6.9 months, and median OS of 15.9 months in previously treated KRAS G12C-mutant metastatic CRC [32,33]. Because this was a single-arm cohort, comparative control-arm outcomes were not available. More recently, dual KRAS G12C and EGFR blockade has entered clinical practice in KRAS G12C-mutant colorectal cancer, with FDA approvals of adagrasib plus cetuximab in 2024 and sotorasib plus panitumumab in 2025 in previously treated metastatic CRC. In CodeBreaK 300, sotorasib plus panitumumab improved PFS compared with investigator’s choice therapy in previously treated KRAS G12C-mutant metastatic CRC. In the approved 960 mg sotorasib plus panitumumab arm, median PFS was 5.6 months compared with 2.2 months in the investigator’s choice arm, and ORR was 26% versus 0%, respectively. The final OS analysis showed a favorable but statistically non-significant trend [28,34] (Table 2B).

### 5.4. Emerging Next-Generation G12C Inhibitors

Despite the success of first-generation KRAS G12C inhibitors, acquired resistance and adaptive signaling remain major clinical challenges [26,27,35,36]. Consequently, several next-generation KRAS G12C inhibitors with improved potency, pharmacokinetic properties, and CNS activity are under active development. Divarasib (GDC-6036) has demonstrated potent and selective KRAS G12C inhibition with encouraging early clinical activity and favorable tolerability profiles [37,38]. Other emerging agents include glecirasib [39,40], olomorasib [41,42], and D-1553 [43,44], many of which are being evaluated in combination with EGFR, SHP2, SOS1, MEK inhibitors, and immunotherapy to overcome adaptive resistance mechanisms. JDQ443 (opnurasib), a covalent KRAS G12C inhibitor previously evaluated in the KontRASt clinical trial program, demonstrated preliminary antitumor activity in early-phase studies [45,46]. However, development of JDQ443 was subsequently discontinued following strategic reassessment by Novartis. Representative KRAS G12C inhibitors and their clinical development status are summarized in Table 1. Collectively, the rapid evolution of KRAS G12C inhibitors has established the foundation for broader KRAS-targeted therapeutic strategies, including KRAS G12D inhibitors, RAS(ON)-targeted agents, and pan-RAS inhibition.

### 5.5. Current Clinical Use of Approved KRAS Inhibitors

At present, the clinical use of approved KRAS inhibitors is largely limited to KRAS G12C-mutated advanced cancers. In NSCLC, sotorasib and adagrasib are approved as single agents for patients with locally advanced or metastatic KRAS G12C-mutated disease who have received at least one prior systemic therapy. Thus, these agents are primarily used in the second-line or later setting rather than as first-line standard therapy.

In metastatic colorectal cancer, KRAS G12C inhibitors are used in combination with EGFR antibodies after prior standard chemotherapy. Sotorasib plus panitumumab and adagrasib plus cetuximab are approved for KRAS G12C-mutated metastatic colorectal cancer after prior fluoropyrimidine-, oxaliplatin-, and irinotecan-based chemotherapy. In contrast, KRAS G12D inhibitors, pan-RAS/RAS(ON) inhibitors, and KRAS degraders remain investigational and are not yet established as routine clinical therapies.

Clinical experience with KRAS G12C inhibitors established OFF-state trapping as a valid therapeutic principle. However, efficacy varies by tumor lineage: NSCLC is relatively sensitive to monotherapy, whereas CRC often requires EGFR blockade due to upstream feedback reactivation. Thus, G12C inhibition demonstrates that allele-specific targeting must be interpreted together with nucleotide-state accessibility, tumor context, and adaptive signaling.

## 6. Mechanisms of Resistance to KRAS Inhibitors

**Figure 2:**
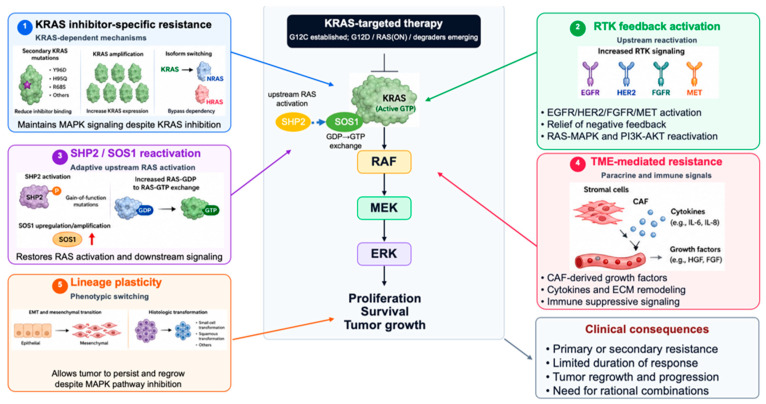
Mechanisms of resistance and adaptive signaling in KRAS-targeted therapy. KRAS-targeted therapy can be limited by on-target resistance, adaptive pathway reactivation, tumor microenvironment-mediated resistance, and non-genetic lineage plasticity. On-target mechanisms include secondary *KRAS* mutations, *KRAS* amplification, and activation of alternative RAS isoforms, which maintain MAPK signaling despite KRAS inhibition. Off-target mechanisms include feedback activation of receptor tyrosine kinases such as EGFR, HER2, FGFR, and MET, as well as SHP2-SOS1-mediated upstream RAS reactivation. Tumor microenvironmental factors, including CAF-derived growth factors, cytokines, extracellular matrix remodeling, and immune-suppressive signaling, may further promote resistance. Lineage plasticity and histologic transformation can reduce KRAS–MAPK dependency and allow tumor persistence. These mechanisms underlie primary and acquired resistance and support the rationale for biomarker-guided combination strategies.

Although KRAS G12C inhibitors have demonstrated significant clinical activity, therapeutic resistance frequently emerges through both on-target and off-target mechanisms. Similar to other targeted therapies, KRAS inhibition induces adaptive signaling rewiring, clonal evolution, and activation of bypass pathways. The major mechanisms of resistance and adaptive signaling during KRAS-targeted therapy are illustrated in Figure 2.

### 6.1. KRAS Inhibitor-Specific Resistance Mechanisms

KRAS inhibitor-specific resistance involves alterations affecting KRAS itself, including secondary mutations within or adjacent to the switch-II pocket such as R68S, H95D/Q/R, Y96C/D, and Q99L. These mutations impair inhibitor binding and restore downstream signaling. *KRAS* amplification has also been reported as a resistance mechanism that enhances MAPK pathway output despite continued KRAS inhibition [35,36,47]. Importantly, multiple resistance alterations may emerge simultaneously during treatment, highlighting the heterogeneous and dynamic nature of KRAS inhibitor resistance.

### 6.2. General Adaptive Resistance Mechanisms Shared with Targeted Therapies

Broader adaptive resistance mechanisms to KRAS inhibitors resemble those observed with other targeted therapies, such as EGFR or ALK inhibitors in lung cancer [48,49]. These include bypass RTK activation, secondary pathway reactivation, gene amplification, phenotypic plasticity, and microenvironment-mediated survival signaling. In colorectal cancer, EGFR-mediated feedback activation is a major mechanism limiting the efficacy of KRAS G12C inhibitor monotherapy and provides a rationale for combined KRAS and EGFR inhibition [28,32]. Upstream RTK signaling can also reactivate RAS through SHP2- and SOS1-dependent nucleotide exchange, thereby restoring MAPK signaling despite KRAS inhibition. This provides a rationale for combination strategies targeting SHP2 or SOS1, although sensitivity to these approaches may vary according to *KRAS* allele, nucleotide-state dependence, and tumor lineage. Additional bypass mechanisms include SHP2-mediated signaling, activation of NRAS/BRAF/MEK/ERK pathways, and MET amplification [27,50]. These adaptive responses can restore MAPK signaling independently of KRAS G12C activity and support the need for rational combination strategies.

### 6.3. Non-Genetic, Lineage, and Tumor Microenvironment-Mediated Resistance

Non-genetic mechanisms also contribute to resistance and are not unique to KRAS inhibitors. Epithelial-to-mesenchymal transition (EMT), lineage plasticity, and histologic transformation can reduce KRAS dependency and promote alternative survival signaling pathways [35,36].

In addition, the tumor microenvironment plays an important role in adaptive resistance through stromal remodeling, inflammatory cytokines, extracellular matrix interactions, and immune suppressive signaling. Collectively, these findings indicate that resistance to KRAS-targeted therapy is driven by a complex interplay between KRAS-specific genomic evolution, general signaling adaptation, phenotypic plasticity, and tumor microenvironmental dynamics [12].

## 7. Beyond G12C: KRAS G12D and Other Allele-Specific Strategies

Although KRAS G12C inhibitors established proof-of-concept for direct KRAS targeting, many KRAS-driven cancers, particularly pancreatic ductal adenocarcinoma (PDAC), are dominated by non-G12C alterations such as KRAS G12D and G12V. Consequently, substantial efforts have shifted toward broader allele-specific therapeutic strategies.

### 7.1. Biological Importance of KRAS G12D

*KRAS* G12D is one of the most common *KRAS* mutations and represents the predominant *KRAS* allele in PDAC [1,14,15]. Compared with *KRAS* G12C, *KRAS* G12D more strongly favors the active GTP-bound state and promotes sustained MAPK signaling. In PDAC, KRAS G12D also contributes to stromal remodeling, immune suppression, and metabolic reprogramming, highlighting its central role in pancreatic tumor biology [15,51,52]. Because PDAC exhibits profound dependence on KRAS signaling, KRAS G12D has emerged as a particularly attractive therapeutic target.

### 7.2. MRTX1133 and Emerging G12D Inhibitors

Mechanistically, KRAS G12D differs from KRAS G12C in ways that directly affect drug design. KRAS G12C retains sufficient nucleotide cycling to allow covalent inhibitors to trap the GDP-bound OFF state through the mutant cysteine residue. In contrast, KRAS G12D lacks a covalent cysteine handle and exhibits distinct electrostatic and conformational features around the switch-II region. Therefore, G12D inhibition requires non-covalent or active-state strategies that recognize mutation-specific structural features rather than relying on irreversible covalent binding. This distinction explains why G12D targeting represents a pharmacologic strategy that is mechanistically different from G12C OFF-state trapping. MRTX1133 represented a direct KRAS G12D inhibition as one of the first highly selective non-covalent KRAS G12D inhibitors [14,15]. Preclinical studies demonstrated potent suppression of KRAS G12D signaling and marked antitumor activity in pancreatic and colorectal cancer models [51,53]. Structural analyses further showed that MRTX1133 could stabilize an inactive KRAS G12D conformation despite the absence of a targetable cysteine residue.

However, the clinical trial of MRTX1133 was terminated because of formulation challenges, according to the clinical trial registry [54] (NCT05737706). Nevertheless, MRTX1133 provided important proof-of-concept for direct KRAS G12D inhibition and established the feasibility of targeting non-G12C KRAS alleles. Subsequent efforts have accelerated development of next-generation KRAS G12D inhibitors. Among these, zoldonrasib (RMC-9805) has emerged as an investigational RAS(ON)-selective KRAS G12D inhibitor currently under early clinical evaluation, with preliminary activity reported in pancreatic ductal adenocarcinoma [55] (NCT06040541). Additional KRAS G12D-targeted agents, including HRS-4642 and VS-7375, are also undergoing early clinical evaluation. Collectively, these developments highlight the rapid evolution of KRAS G12D-targeted therapy and the continued expansion of allele-specific therapeutic strategies beyond KRAS G12C.

### 7.3. Other Allele-Specific Approaches

Therapeutic approaches targeting KRAS G12V, G13D, and codon 61 mutations are also under investigation. KRAS G12V remains challenging because of its stable active-state conformation, whereas KRAS G13D exhibits distinct biochemical behavior that may influence EGFR inhibitor sensitivity in colorectal cancer [4,12,56]. Codon 61 mutations such as Q61H strongly impair intrinsic GTP hydrolysis and exhibit relative independence from upstream SOS1/SHP2 regulation, limiting sensitivity to certain upstream-targeted therapies [13,16]. In addition to small-molecule inhibitors, alternative approaches including neoantigen vaccines, adoptive T-cell therapies, RNA-targeted therapeutics, and KRAS degraders are actively being explored [10,12].

### 7.4. Challenges in Non-G12C KRAS Targeting

Unlike KRAS G12C inhibitors, which exploit a reactive cysteine residue for covalent binding, direct targeting of KRAS G12D presents substantially greater pharmacologic challenges. KRAS G12C inhibitors such as sotorasib and adagrasib selectively bind a transient switch-II pocket (S-IIP) adjacent to the switch II region and irreversibly trap KRAS in its inactive GDP-bound state through covalent interaction with Cys12 residue [5,6,7,20,21,23,29]. Importantly, KRAS G12C retains substantial intrinsic nucleotide-cycling capacity despite its oncogenic activity, thereby allowing intermittent access to the GDP-bound conformation required for inhibitor engagement. In contrast, KRAS G12D lacks a targetable cysteine residue for covalent inhibition and more strongly favors the active GTP-bound state, thereby reducing accessibility to inactive-state selective inhibitors. These biochemical differences necessitated the development of fundamentally different therapeutic strategies for KRAS G12D. Rather than relying on irreversible covalent trapping, KRAS G12D inhibitors such as MRTX1133 were designed as highly selective non-covalent inhibitors capable of recognizing mutation-specific structural and electrostatic features within the switch-II region. Structural studies demonstrated that MRTX1133 stabilizes an inactive-like conformation of KRAS G12D despite the absence of covalent interaction [14,15]. The limitations of GDP-state selective inhibition have also accelerated development of RAS(ON)-targeted therapies designed to directly inhibit active GTP-bound KRAS conformations [12]. This transition from OFF-state trapping toward ON-state targeting represents a major conceptual shift in modern KRAS drug discovery.

KRAS G12D shows that non-G12C targeting cannot simply replicate the G12C model. The absence of a covalent cysteine handle and distinct nucleotide-state behavior requires different pharmacologic strategies. MRTX1133 provided preclinical proof of concept for non-covalent G12D inhibition, but its clinical discontinuation highlights the translational challenges of potency, formulation, pharmacokinetics, and therapeutic window.

## 8. Pan-RAS and RAS(ON) Therapeutic Strategies

Although allele-specific KRAS inhibitors have transformed the therapeutic landscape of *KRAS*-mutant cancers, several important limitations remain. Current mutation-specific inhibitors target only selected *KRAS* alleles, and therapeutic resistance frequently emerges through secondary *KRAS* alterations, bypass signaling activation, and adaptive pathway reprogramming. In addition, many clinically relevant *KRAS* mutations, including G12V and Q61 variants, remain difficult to target directly. These limitations have driven substantial interest in broader therapeutic approaches capable of suppressing multiple RAS isoforms, multiple *KRAS* alleles, or active-state RAS signaling itself.

Recent advances in structural biology, protein chemistry, and molecular pharmacology have therefore accelerated the development of pan-RAS and RAS(ON)-targeted therapeutic strategies designed to overcome the constraints of allele-specific inhibition.

### 8.1. Concept of Pan-RAS Inhibition

Pan-RAS inhibition refers to therapeutic strategies capable of suppressing signaling across multiple RAS isoforms or multiple oncogenic *KRAS* alleles simultaneously [10,12]. Unlike allele-specific inhibitors that selectively target individual mutant residues such as G12C or G12D, pan-RAS approaches aim to broadly inhibit RAS signaling irrespective of the specific mutational context.

BI-2865 is a representative direct pan-KRAS inhibitor that illustrates the feasibility of broad KRAS targeting beyond single-allele inhibition [57]. It binds inactive-state KRAS in a non-covalent manner across multiple KRAS variants, including common oncogenic mutants. Although BI-2865 remains preclinical, it provides important proof-of-concept for direct pan-KRAS inhibition.

One major conceptual advance in this field has been the development of active-state targeting strategies. Whereas first-generation KRAS G12C inhibitors preferentially bind the inactive GDP-bound state, many oncogenic *KRAS* mutants strongly favor the active GTP-bound conformation. This limitation has motivated the development of RAS(ON) inhibitors that selectively engage active-state RAS proteins [10,12].

RAS(ON) and pan-RAS inhibitors address a different conformational state from conventional G12C inhibitors. Whereas G12C inhibitors preferentially bind inactive GDP-bound KRAS, RAS(ON) inhibitors are designed to engage active GTP-bound RAS conformations that drive effector binding to RAF, PI3K, and other downstream pathways. This may expand allele coverage but also raises concerns regarding wild-type RAS inhibition, therapeutic window, and adaptive resistance. RAS(ON) inhibitors are particularly attractive because they may overcome several resistance mechanisms associated with GDP-state selective inhibition, including adaptive nucleotide cycling and secondary mutations that alter inactive-state accessibility. Furthermore, active-state targeting may enable therapeutic inhibition of *KRAS* alleles that exhibit persistent GTP loading and reduced dependence on upstream regulatory signaling.

In parallel, multi-selective inhibition strategies have emerged to suppress broader RAS network signaling. These approaches may target multiple *KRAS* alleles, wild-type RAS isoforms contributing to bypass signaling, or shared structural vulnerabilities across RAS family proteins. Such strategies are especially relevant given the extensive signaling plasticity and pathway redundancy observed in KRAS-driven cancers.

Importantly, pan-RAS inhibition also carries potential therapeutic risks, including toxicity associated with suppression of physiologic wild-type RAS signaling. Therefore, achieving an optimal therapeutic window remains a major challenge in pan-RAS drug development.

### 8.2. RMC-6236 and Related Agents

Among emerging pan-RAS therapeutics, RMC-6236 has become one of the most prominent and clinically advanced agents [58]. RMC-6236 is a RAS(ON) multi-selective inhibitor designed to target active GTP-bound RAS proteins across multiple *KRAS* mutant alleles. Unlike allele-specific covalent inhibitors, RMC-6236 exploits active-state RAS conformations and forms high-affinity complexes capable of suppressing downstream signaling activity.

Early clinical studies demonstrated encouraging antitumor activity across multiple *KRAS*-mutant solid tumors, including pancreatic cancer, colorectal cancer, and NSCLC. Particularly notable activity has been observed in PDAC, a disease in which therapeutic options remain limited and *KRAS* mutations are nearly universal. The emergence of clinically meaningful activity in heavily pretreated *KRAS*-mutant pancreatic cancer patients has generated substantial enthusiasm regarding the therapeutic potential of pan-RAS inhibition.

Clinical development of pan-RAS and RAS(ON) inhibitors has advanced to randomized evaluation, particularly in pancreatic ductal adenocarcinoma (PDAC), a disease characterized by frequent oncogenic RAS pathway activation. The global phase III RASolute 302 trial evaluated daraxonrasib (RMC-6236), an oral multi-selective RAS(ON) inhibitor, versus investigator’s choice chemotherapy in patients with previously treated metastatic PDAC. In this trial, daraxonrasib significantly improved overall survival and progression-free survival compared with chemotherapy. In the overall population, median overall survival was 13.2 months with daraxonrasib versus 6.7 months with chemotherapy, and median progression-free survival was 7.2 versus 3.6 months, respectively. Similar benefits were observed in the RAS G12 population [59]. These data provide clinical proof-of-concept that direct inhibition of active RAS signaling can produce meaningful therapeutic benefit in KRAS/RAS-driven PDAC. However, further follow-up and regulatory evaluation are needed to define its eventual role in clinical practice.

Additional RAS(ON) and pan-RAS inhibitors are also under active development. These include structurally diverse compounds designed to enhance potency, improve selectivity, optimize pharmacokinetic properties, and overcome resistance-associated conformational changes. Collectively, these agents represent a major next-generation expansion of KRAS-targeted therapy beyond single-allele inhibition.

### 8.3. SHP2 and SOS1 Inhibition

In addition to direct RAS inhibitors, indirect suppression of RAS signaling through SHP2 and SOS1 inhibition has emerged as an important therapeutic strategy. SHP2 and SOS1 function upstream of KRAS and play central roles in receptor tyrosine kinase-mediated RAS activation [16,60].

SHP2 is a protein tyrosine phosphatase that promotes RAS signaling through multiple mechanisms, including facilitation of SOS1-mediated nucleotide exchange and regulation of KRAS conformational dynamics through dephosphorylation of Tyr32 and Tyr64 [8,9]. Pharmacologic inhibition of SHP2 suppresses RTK-mediated RAS activation and has demonstrated preclinical activity in KRAS-driven cancers [16,26].

Importantly, SHP2 inhibition has emerged as a particularly attractive combination strategy with KRAS inhibitors because it suppresses adaptive feedback reactivation of MAPK signaling. Inhibition of KRAS G12C often results in compensatory activation of upstream RTK signaling and wild-type RAS isoforms, processes that may be attenuated through concurrent SHP2 blockade.

Similarly, SOS1 inhibitors impair nucleotide exchange and reduce conversion of inactive GDP-bound KRAS to the active GTP-bound state. Because KRAS G12C inhibitors preferentially target the GDP-bound conformation, combined inhibition of SOS1 may enhance therapeutic efficacy by increasing the fraction of inhibitor-accessible KRAS [60].

Multiple SHP2 and SOS1 inhibitors are currently undergoing clinical evaluation in combination with KRAS-targeted therapies. However, mutation-specific biological differences remain important. For example, KRAS Q61H exhibits marked resistance to SHP2-mediated regulation, thereby limiting sensitivity to SHP2 inhibition [13,50]. These findings further underscore the importance of mutation-specific KRAS biology in therapeutic development.

### 8.4. RAS Degraders and Novel Modalities

Beyond conventional inhibition strategies, several highly innovative therapeutic modalities are emerging to target KRAS and broader RAS signaling networks. Among these approaches, targeted protein degradation strategies have generated particularly strong interest.

PROTAC (proteolysis-targeting chimera) technologies are designed to induce selective degradation of KRAS proteins through recruitment of E3 ubiquitin ligases and subsequent proteasomal destruction [61,62,63,64]. Unlike occupancy-driven inhibition, degraders may achieve sustained suppression of oncogenic signaling through elimination of the target protein itself. KRAS degraders targeting specific mutant alleles or broader RAS conformations are under active development, although most remain at the preclinical stage. Molecular glue approaches represent another distinct preclinical strategy. These compounds stabilize novel protein–protein interactions that promote selective degradation or functional inactivation of KRAS. Similarly, tri-complex inhibitors have emerged as highly innovative therapeutic platforms capable of simultaneously engaging KRAS and additional intracellular binding partners to stabilize inactive signaling complexes.

Additional emerging modalities include RNA-targeted therapeutics, antisense oligonucleotides, siRNA-based suppression [65,66], neoantigen vaccines [67,68], and adoptive T-cell therapies targeting mutant KRAS-derived epitopes [69,70]. These modalities vary substantially in clinical maturity, ranging from preclinical degrader platforms to early-phase clinical studies of RNA-based or vaccine approaches.

In addition to small-molecule inhibitors and degraders, several emerging modalities are being explored for KRAS-targeted therapy. These include DARPins [71], cyclic peptides [72], KRAS–RAF protein–protein interaction inhibitors [73], and KRAS–GAP molecular glue-based approaches [74]. These strategies aim to overcome the limitations of conventional small-molecule inhibition by targeting KRAS conformations, effector interactions, or regulatory protein complexes. However, most remain preclinical or early-stage, and their clinical utility will depend on further validation of selectivity, intracellular delivery, pharmacokinetics, and therapeutic window.

Representative pan-RAS, RAS pathway-targeted, and degradation-based therapeutic approaches are summarized in Table 3. Collectively, these next-generation approaches highlight the rapidly expanding therapeutic landscape of KRAS-targeted oncology. The field is now transitioning from single-mutation inhibition toward integrated suppression of dynamic RAS signaling networks through structurally diverse and mechanistically innovative therapeutic strategies.

Pan-RAS and RAS(ON) strategies shift KRAS targeting from single-allele inhibition toward broader suppression of active RAS signaling. Their potential advantage is wider allele coverage, but their key limitation is the need to preserve a therapeutic window despite possible wild-type RAS inhibition and adaptive resistance.

## 9. Biomarkers and Precision Oncology Approaches

As KRAS-targeted therapies expand from G12C inhibitors to G12D, pan-RAS, and combination strategies, biomarker-driven patient selection and longitudinal molecular monitoring are becoming increasingly important. *KRAS* mutation status alone is no longer sufficient to define therapeutic vulnerability. Instead, precision treatment of *KRAS*-mutant cancers requires integration of *KRAS* allele type, tumor lineage, co-occurring genomic alterations, adaptive signaling context, and dynamic changes during therapy.

### 9.1. Genomic Biomarkers

Co-occurring genomic alterations substantially influence the biology and therapeutic responsiveness of *KRAS*-mutant cancers. In NSCLC, alterations in *STK11*, *KEAP1*, and *TP53* define biologically distinct molecular subsets with different immune phenotypes and treatment outcomes [75,76]. *KRAS*-mutant tumors with *TP53* co-mutation often show higher inflammatory signaling and may be more responsive to immune checkpoint blockade. In contrast, *STK11* and *KEAP1* alterations are frequently associated with immune-cold tumor microenvironments, metabolic reprogramming, oxidative stress adaptation, and poorer clinical outcomes.

In colorectal cancer, tumor lineage and pathway context are especially important. *KRAS*-mutant CRC frequently exhibits strong dependency on EGFR-mediated feedback signaling, particularly after KRAS G12C inhibition [26,27,28,32,33,56]. Additional alterations in *APC*, *TP53*, *PIK3CA*, and receptor tyrosine kinase pathways may further shape treatment response and resistance. Therefore, *KRAS* allele status should be interpreted together with broader pathway-level genomic context.

In PDAC, *KRAS* mutations almost always occur within a complex genomic background that commonly includes alterations in *TP53*, *CDKN2A*, and *SMAD4*. Among these, *SMAD4* loss is associated with aggressive disease biology, metastatic potential, and altered tumor microenvironmental features [52]. These co-mutations may influence sensitivity to KRAS G12D or pan-RAS inhibitors and will likely become important stratification factors in future clinical trials.

Importantly, the same *KRAS* allele can behave differently depending on tumor lineage and co-mutational context. For example, KRAS G12C inhibition produces substantially different outcomes in NSCLC and CRC, largely due to lineage-specific feedback regulation [23,26,27,28,32]. This highlights the need to move beyond single-gene biomarker selection toward integrated genomic and pathway-based precision oncology.

### 9.2. ctDNA and Liquid Biopsy

Circulating tumor DNA has emerged as a powerful tool for monitoring *KRAS*-mutant cancers throughout the disease course. Because *KRAS* mutations are often clonal founder events, mutant *KRAS* ctDNA can serve as a highly informative molecular marker for tumor burden, treatment response, minimal residual disease, and emergence of resistance [29,47,77,78].

In advanced disease, serial ctDNA analysis can provide early evidence of molecular response to KRAS-targeted therapy. Declines in *KRAS*-mutant allele fraction after treatment initiation may precede radiographic response and could help identify patients likely to derive durable benefit [47]. Conversely, early persistence or rebound of *KRAS*-mutant ctDNA may indicate incomplete pathway suppression or emerging resistance.

ctDNA is also particularly valuable for detecting acquired resistance mechanisms. During KRAS inhibitor therapy, liquid biopsy can reveal secondary *KRAS* mutations, *KRAS* amplification, activation of bypass pathways, and emergence of heterogeneous resistant subclones. Because multiple resistance mechanisms can coexist within a single patient, ctDNA offers a broader real-time view of tumor evolution than single-site tissue biopsy.

In earlier-stage disease, ctDNA-based minimal residual disease assessment may enable risk stratification after surgery or definitive therapy [47,79]. For *KRAS*-mutant cancers such as CRC, PDAC, and potentially head and neck cancers [80], postoperative ctDNA detection may identify patients at high risk of recurrence and guide adjuvant therapeutic intensification. As KRAS-targeted therapies move into perioperative settings, ctDNA could become central to selecting patients for adjuvant KRAS inhibition or adaptive treatment escalation.

Liquid biopsy may also facilitate clinical trial design by enabling molecular eligibility screening, pharmacodynamic monitoring, and early assessment of resistance. Integration of ctDNA with tissue-based genomic profiling, transcriptomics, and immune biomarkers will likely improve precision treatment strategies for *KRAS*-mutant cancers.

### 9.3. Tumor Heterogeneity and Adaptive Evolution

*KRAS*-mutant cancers are characterized by marked intertumoral and intratumoral heterogeneity. Although *KRAS* mutations often function as early clonal driver events, downstream signaling dependencies, co-mutation patterns, tumor microenvironmental states, and resistance trajectories vary substantially across patients and tumor types.

Therapeutic pressure from KRAS inhibition can rapidly reshape tumor clonal architecture [35,36,47,81]. Sensitive clones may regress, whereas pre-existing resistant subclones or newly acquired resistant populations expand. This adaptive evolution can involve secondary *KRAS* mutations, activation of alternative *RAS* isoforms, receptor tyrosine kinase upregulation, MAPK pathway reactivation, lineage plasticity, and immune escape.

Tumor heterogeneity is particularly relevant for pan-RAS and combination strategies. While broader pathway inhibition may suppress multiple KRAS-driven clones, it may also impose selective pressure favoring non-RAS-dependent survival programs. Therefore, durable disease control will likely require adaptive therapeutic strategies based on longitudinal molecular monitoring.

A future precision oncology framework for *KRAS*-mutant cancers should therefore integrate baseline genomic profiling with serial ctDNA analysis and dynamic assessment of treatment-induced evolution [47,77,78,79]. Rather than treating *KRAS*-mutant cancer as a static molecular category, clinicians and investigators should conceptualize KRAS-driven tumors as evolving signaling ecosystems shaped by allele-specific biology, tissue lineage, therapeutic pressure, and microenvironmental context.

**Figure 3:**
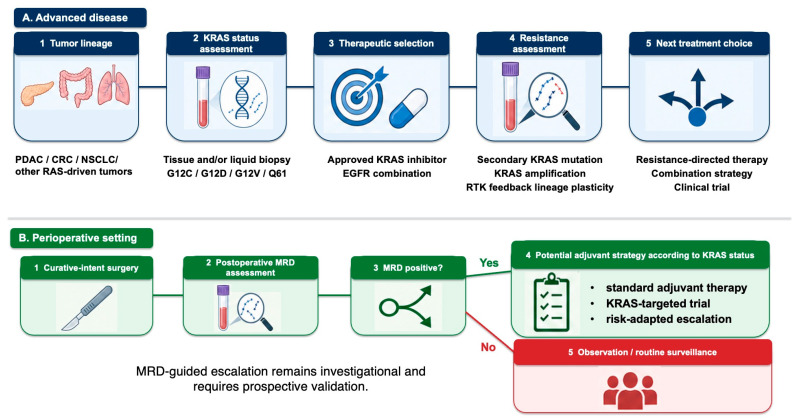
Biomarker-guided framework for KRAS-targeted therapy in advanced and perioperative settings. In advanced disease, tumor lineage and clinical context guide *KRAS* status assessment using tissue and/or liquid biopsy, followed by therapeutic selection according to *KRAS* allele, tumor type, and available treatment options. Serial liquid biopsy and ctDNA monitoring may support molecular response assessment and detection of emerging resistance mechanisms, which may inform subsequent resistance-directed therapy or clinical trial selection. In the perioperative setting, postoperative ctDNA-based MRD assessment may identify patients at increased recurrence risk; however, MRD-guided escalation, *KRAS*-directed adjuvant treatment, and perioperative ctDNA-guided adaptation remain investigational and require prospective validation. ctDNA and tissue biopsy are complementary, with ctDNA enabling serial molecular monitoring and tissue biopsy providing histologic and microenvironmental information.

This biomarker-driven approach may be especially important as KRAS-targeted therapies move beyond metastatic disease into earlier treatment settings, including neoadjuvant, adjuvant, and molecular residual disease-directed strategies (Figure 3).

Thus, biomarker-guided KRAS therapy should be interpreted according to evidence maturity. *KRAS* allele status is already clinically actionable, whereas co-mutations, tumor lineage, and serial ctDNA dynamics remain variably validated. ctDNA is useful for monitoring response and resistance, but ctDNA-guided treatment switching or perioperative escalation remains investigational.

## 10. Future Perspectives and Conclusions

KRAS-targeted therapy has moved beyond the initial G12C paradigm toward non-G12C, RAS(ON), pan-RAS, and degradation-based strategies. The clinical success of sotorasib and adagrasib established direct KRAS inhibition as a feasible therapeutic principle, but also revealed important limitations, including adaptive pathway reactivation, tumor-lineage dependence, acquired resistance, and limited durability of response.

The next phase of KRAS precision oncology should therefore focus on matching therapeutic strategy to molecular context. Allele-specific inhibitors, pan-RAS/RAS(ON) agents, degraders, RNA-targeted therapies, and immunotherapeutic strategies differ substantially in evidence maturity, target state, allele coverage, therapeutic window, and resistance liabilities. Their clinical roles should be defined through prospective trials that incorporate tumor lineage, co-mutations, baseline pathway activity, and molecular response assessment.

Biomarker-guided treatment will be central to this framework, but its current utility remains uneven. *KRAS* allele status is clinically actionable, whereas ctDNA-guided treatment switching, MRD-based escalation, and perioperative adaptation remain investigational. These strategies require prospective validation before routine clinical implementation.

In conclusion, the post-G12C era requires a shift from single-agent, allele-specific targeting toward a state-selective, lineage-aware, resistance-informed, and biomarker-guided framework. Progress will depend not only on developing more potent KRAS-directed agents, but also on aligning each strategy with the appropriate tumor biology and level of supporting evidence. 

## Figures and Tables

**Table 1:** Major KRAS G12C Inhibitors.

Drug	Company	Mechanism	Key Features	Clinical Status	Evidence Level
Sotorasib	Amgen (Thousand Oaks, CA, USA)	Covalent KRAS G12C inhibitor	First approved KRAS inhibitor	Approved	1
Adagrasib	Bristol Myers Squibb (formerly Mirati Therapeutics) (New York, NY, USA)	Covalent KRAS G12C inhibitor	CNS activity	Approved	1
Divarasib	Roche/Genentech (Basel, Switzerland)	Covalent KRAS G12C inhibitor	High potency/selectivity	Clinical development	2
Glecirasib	Jacobio (Beijing, China)	Covalent KRAS G12C inhibitor	Emerging global data	Clinical development	2–3
Olomorasib	Eli Lilly (Indianapolis, IN, USA)	Covalent KRAS G12C inhibitor	Improved pharmacokinetics	Clinical development	2–3
JDQ443 (opnurasib)	Novartis (Basel, Switzerland)	Covalent KRAS G12C inhibitor	Evaluated in KontRASt trials	Development discontinued	2–3
Garsorasib (D-1553)	InventisBio/Betta (Shanghai, China)	Covalent KRAS G12C inhibitor	Emerging CRC and pancreatic cancer data	Clinical development	2–3

**Table 2:** Comparative clinical evidence for KRAS G12C-targeted therapy (**A**,**B**).

**A. KRAS G12C NSCLC** **:** **Sotorasib vs. Adagrasib**							
**Agent/Trial**	**Trial**	**Design**	***n***	**Comparator**	**ORR**	**PFS**	**OS**
Sotorasib	CodeBreaK 100	Phase II, single-arm	126	None	41%	6.3 mo	12.5 mo
Sotorasib	CodeBreaK 200	Phase III, randomized	345	Docetaxel	28.1% vs. 13.2%	5.6 vs. 4.5 mo	10.6 vs. 11.3 mo
Adagrasib	KRYSTAL-1	Phase II, single-arm	116	None	42.90%	6.5 mo	12.6 mo
Adagrasib	KRYSTAL-12	Phase III, randomized	453	Docetaxel	32% vs. 9%	5.5 vs. 3.8 mo	Immature
**B. KRAS G12C CRC:Monotherapy vs. EGFR Combination**							
**Strategy**	**Trial**	**Design**	***n***	**Comparator**	**ORR**	**PFS**	**OS**
Sotorasib monotherapy	CodeBreaK 100 CRC	Phase II, single-arm	62	None	9.70%	4.0 mo	10.6 mo
Sotorasib + panitumumab	CodeBreaK 300	Phase III, randomized	53 vs. 54	Investigator’s choice	26.4% vs. 0%	5.6 vs. 2.2 mo	OS trend, not definitive
Adagrasib + cetuximab	KRYSTAL-1 updated CRC cohort	Phase I/II, single-arm	94	None	34%	6.9 mo	15.9 mo

**Table 3:** Pan-RAS and RAS Pathway-Targeted Agents.

Agent	Class	Mechanism	Target	Clinical Status	Evidence Level
Daraxonrasib (RMC-6236)	RAS(ON) inhibitor	Active-state multi-selective inhibition	Multiple KRAS alleles	Phase III (RASolute 302)	1
TNO155	SHP2 inhibitor	RTK-RAS signaling blockade	SHP2	Clinical development	2–3
RMC-4630	SHP2 inhibitor	Adaptive pathway suppression	SHP2	Clinical development	2–3
BI 1701963	SOS1 inhibitor	Blocks SOS1::KRAS interaction	SOS1	Clinical development	2–3
MRTX0902	SOS1 inhibitor	KRAS activation suppression	SOS1	Clinical development	3
KRAS degraders	PROTAC/degrader	Targeted KRAS degradation	KRAS	Preclinical	4
siRNA/ASO agents	RNA-targeted therapy	KRAS expression suppression	KRAS mRNA	Early development	3–4
BI-2865	Direct pan-KRAS inhibitor	Non-covalent inactive-state KRAS binding	Multiple KRAS variants	Preclinical	4

## Data Availability

No new data were created or analyzed in this study. Data sharing is not applicable to this article.

## Abbreviations

The following abbreviations are used in this manuscript:

ASO	Antisense oligonucleotide
CAF	Cancer-associated fibroblast
CNS	Central nervous system
CRC	Colorectal cancer
ctDNA	Circulating tumor DNA
ECM	Extracellular matrix
EGFR	Epidermal growth factor receptor
EMT	Epithelial-to-mesenchymal transition
FDA	U.S. Food and Drug Administration
FTI	Farnesyltransferase inhibitor
GAP	GTPase-activating protein
GDP	Guanosine diphosphate
GEF	Guanine nucleotide exchange factor
GTP	Guanosine triphosphate
HR	Hazard ratio
MAPK	Mitogen-activated protein kinase
MEK	Mitogen-activated protein kinase kinase
mCRC	Metastatic colorectal cancer
MRD	Minimal residual disease
NSCLC	Non-small-cell lung cancer
ORR	Objective response rate
OS	Overall survival
PDAC	Pancreatic ductal adenocarcinoma
PFS	Progression-free survival
PI3K	Phosphoinositide 3-kinase
PROTAC	Proteolysis-targeting chimera
RAF	Rapidly accelerated fibrosarcoma kinase
RAS(ON)	Active-state RAS
RTK	Receptor tyrosine kinase
SHP2	Src homology region 2-containing protein tyrosine phosphatase 2
S-IIP	Switch-II pocket
siRNA	Small interfering RNA
SOS1	Son of sevenless homolog 1
TME	Tumor microenvironment

## References

[1] Prior I.A., Hood F.E., Hartley J.L. (2020). The Frequency of Ras Mutations in Cancer. Cancer Res..

[2] Cox A.D., Fesik S.W., Kimmelman A.C., Luo J., Der C.J. (2014). Drugging the undruggable RAS: Mission possible?. Nat. Rev. Drug Discov..

[3] Kano Y., Cook J.D., Lee J.E., Ohh M. (2016). New structural and functional insight into the regulation of Ras. Semin. Cell Dev. Biol..

[4] Simanshu D.K., Nissley D.V., McCormick F. (2017). RAS Proteins and Their Regulators in Human Disease. Cell.

[5] Ostrem J.M., Peters U., Sos M.L., Wells J.A., Shokat K.M. (2013). K-Ras(G12C) inhibitors allosterically control GTP affinity and effector interactions. Nature.

[6] Patricelli M.P., Janes M.R., Li L.S., Hansen R., Peters U., Kessler L.V., Chen Y., Kucharski J.M., Feng J., Ely T. (2016). Selective Inhibition of Oncogenic KRAS Output with Small Molecules Targeting the Inactive State. Cancer Discov..

[7] Janes M.R., Zhang J., Li L.S., Hansen R., Peters U., Guo X., Chen Y., Babbar A., Firdaus S.J., Darjania L. (2018). Targeting KRAS Mutant Cancers with a Covalent G12C-Specific Inhibitor. Cell.

[8] Kano Y., Gebregiworgis T., Marshall C.B., Radulovich N., Poon B.P.K., St-Germain J., Cook J.D., Valencia-Sama I., Grant B.M.M., Herrera S.G. (2019). Tyrosyl phosphorylation of KRAS stalls GTPase cycle via alteration of switch I and II conformation. Nat. Commun..

[9] Bunda S., Burrell K., Heir P., Zeng L., Alamsahebpour A., Kano Y., Raught B., Zhang Z.Y., Zadeh G., Ohh M. (2015). Inhibition of SHP2-mediated dephosphorylation of Ras suppresses oncogenesis. Nat. Commun..

[10] Moore A.R., Rosenberg S.C., McCormick F., Malek S. (2020). RAS-targeted therapies: Is the undruggable drugged?. Nat. Rev. Drug Discov..

[11] Punekar S.R., Velcheti V., Neel B.G., Wong K.K. (2022). The current state of the art and future trends in RAS-targeted cancer therapies. Nat. Rev. Clin. Oncol..

[12] Hofmann M.H., Gerlach D., Misale S., Petronczki M., Kraut N. (2022). Expanding the Reach of Precision Oncology by Drugging All KRAS Mutants. Cancer Discov..

[13] Gebregiworgis T., Kano Y., St-Germain J., Radulovich N., Udaskin M.L., Mentes A., Huang R., Poon B.P.K., He W., Valencia-Sama I. (2021). The Q61H mutation decouples KRAS from upstream regulation and renders cancer cells resistant to SHP2 inhibitors. Nat. Commun..

[14] Wang X., Allen S., Blake J.F., Bowcut V., Briere D.M., Calinisan A., Dahlke J.R., Fell J.B., Fischer J.P., Gunn R.J. (2022). Identification of MRTX1133, a Noncovalent, Potent, and Selective KRAS^G12D^ Inhibitor. J. Med. Chem..

[15] Hallin J., Bowcut V., Calinisan A., Briere D.M., Hargis L., Engstrom L.D., Laguer J., Medwid J., Vanderpool D., Lifset E. (2022). Anti-tumor efficacy of a potent and selective non-covalent KRAS^G12D^ inhibitor. Nat. Med..

[16] Nichols R.J., Haderk F., Stahlhut C., Schulze C.J., Hemmati G., Wildes D., Tzitzilonis C., Mordec K., Marquez A., Romero J. (2018). RAS nucleotide cycling underlies the SHP2 phosphatase dependence of mutant BRAF-, NF1- and RAS-driven cancers. Nat. Cell Biol..

[17] Rowell C.A., Kowalczyk J.J., Lewis M.D., Garcia A.M. (1997). Direct demonstration of geranylgeranylation and farnesylation of Ki-Ras in vivo. J. Biol. Chem..

[18] Whyte D.B., Kirschmeier P., Hockenberry T.N., Nunez-Oliva I., James L., Catino J.J., Bishop W.R., Pai J.K. (1997). K- and N-Ras are geranylgeranylated in cells treated with farnesyl protein transferase inhibitors. J. Biol. Chem..

[19] Baranyi M., Buday L., Hegedűs B. (2020). K-Ras prenylation as a potential anticancer target. Cancer Metastasis Rev..

[20] Lanman B.A., Allen J.R., Allen J.G., Amegadzie A.K., Ashton K.S., Booker S.K., Chen J.J., Chen N., Frohn M.J., Goodman G. (2020). Discovery of a Covalent Inhibitor of KRAS^G12C^ (AMG 510) for the Treatment of Solid Tumors. J. Med. Chem..

[21] Canon J., Rex K., Saiki A.Y., Mohr C., Cooke K., Bagal D., Gaida K., Holt T., Knutson C.G., Koppada N. (2019). The clinical KRAS(G12C) inhibitor AMG 510 drives anti-tumour immunity. Nature.

[22] Hong D.S., Fakih M.G., Strickler J.H., Desai J., Durm G.A., Shapiro G.I., Falchook G.S., Price T.J., Sacher A., Denlinger C.S. (2020). KRAS^G12C^ Inhibition with Sotorasib in Advanced Solid Tumors. N. Engl. J. Med..

[23] Skoulidis F., Li B.T., Dy G.K., Price T.J., Falchook G.S., Wolf J., Italiano A., Schuler M., Borghaei H., Barlesi F. (2021). Sotorasib for Lung Cancers with *KRAS* p.G12C Mutation. N. Engl. J. Med..

[24] Dy G.K., Govindan R., Velcheti V., Falchook G.S., Italiano A., Wolf J., Sacher A.G., Takahashi T., Ramalingam S.S., Dooms C. (2023). Long-Term Outcomes and Molecular Correlates of Sotorasib Efficacy in Patients With Pretreated KRAS G12C-Mutated Non-Small-Cell Lung Cancer: 2-Year Analysis of CodeBreaK 100. J. Clin. Oncol..

[25] de Langen A.J., Johnson M.L., Mazieres J., Dingemans A.C., Mountzios G., Pless M., Wolf J., Schuler M., Lena H., Skoulidis F. (2023). Sotorasib versus docetaxel for previously treated non-small-cell lung cancer with *KRAS*^G12C^ mutation: A randomised, open-label, phase 3 trial. Lancet.

[26] Ryan M.B., Fece de la Cruz F., Phat S., Myers D.T., Wong E., Shahzade H.A., Hong C.B., Corcoran R.B. (2020). Vertical Pathway Inhibition Overcomes Adaptive Feedback Resistance to *KRAS*^G12C^ Inhibition. Clin. Cancer Res..

[27] Amodio V., Yaeger R., Arcella P., Cancelliere C., Lamba S., Lorenzato A., Arena S., Montone M., Mussolin B., Bian Y. (2020). EGFR Blockade Reverts Resistance to KRAS^G12C^ Inhibition in Colorectal Cancer. Cancer Discov..

[28] Fakih M.G., Salvatore L., Esaki T., Modest D.P., Lopez-Bravo D.P., Taieb J., Karamouzis M.V., Ruiz-Garcia E., Kim T.W., Kuboki Y. (2023). Sotorasib plus Panitumumab in Refractory Colorectal Cancer with Mutated KRAS G12C. N. Engl. J. Med..

[29] Jänne P.A., Riely G.J., Gadgeel S.M., Heist R.S., Ou S.I., Pacheco J.M., Johnson M.L., Sabari J.K., Leventakos K., Yau E. (2022). Adagrasib in Non-Small-Cell Lung Cancer Harboring a KRAS^G12C^ Mutation. N. Engl. J. Med..

[30] Negrao M.V., Spira A.I., Heist R.S., Jänne P.A., Pacheco J.M., Weiss J., Gadgeel S.M., Velastegui K., Yang W., Der-Torossian H. (2023). Intracranial Efficacy of Adagrasib in Patients From the KRYSTAL-1 Trial With *KRAS^G12C^*-Mutated Non-Small-Cell Lung Cancer Who Have Untreated CNS Metastases. J. Clin. Oncol..

[31] Bernstein E., Luo J., Wang K., Negrao M.V., Jänne P.A., Sabari J.K. (2024). Safety and Intracranial Activity of Adagrasib in Patients With *KRAS*^G12C^-Mutated Non-Small-Cell Lung Cancer and Untreated CNS Metastases in the KRYSTAL-1 Trial: A Case Series. JCO Precis. Oncol..

[32] Yaeger R., Weiss J., Pelster M.S., Spira A.I., Barve M., Ou S.I., Leal T.A., Bekaii-Saab T.S., Paweletz C.P., Heavey G.A. (2023). Adagrasib with or without Cetuximab in Colorectal Cancer with Mutated KRAS G12C. N. Engl. J. Med..

[33] Yaeger R., Uboha N.V., Pelster M.S., Bekaii-Saab T.S., Barve M., Saltzman J., Sabari J.K., Peguero J.A., Paulson A.S., Jänne P.A. (2024). Efficacy and Safety of Adagrasib plus Cetuximab in Patients with *KRAS*^G12C^-Mutated Metastatic Colorectal Cancer. Cancer Discov..

[34] Pietrantonio F., Salvatore L., Esaki T., Modest D.P., Lopez-Bravo D.P., Taieb J., Karamouzis M.V., Ruiz-Garcia E., Kim T.W., Kuboki Y. (2025). Overall Survival Analysis of the Phase III CodeBreaK 300 Study of Sotorasib Plus Panitumumab Versus Investigator’s Choice in Chemorefractory *KRAS* G12C Colorectal Cancer. J. Clin. Oncol..

[35] Awad M.M., Liu S., Rybkin I.I., Arbour K.C., Dilly J., Zhu V.W., Johnson M.L., Heist R.S., Patil T., Riely G.J. (2021). Acquired Resistance to KRAS^G12C^ Inhibition in Cancer. N. Engl. J. Med..

[36] Zhao Y., Murciano-Goroff Y.R., Xue J.Y., Ang A., Lucas J., Mai T.T., Da Cruz Paula A.F., Saiki A.Y., Mohn D., Achanta P. (2021). Diverse alterations associated with resistance to KRAS(G12C) inhibition. Nature.

[37] Desai J., Alonso G., Kim S.H., Cervantes A., Karasic T., Medina L., Shacham-Shmueli E., Cosman R., Falcon A., Gort E. (2024). Divarasib plus cetuximab in *KRAS G12C*-positive colorectal cancer: A phase 1b trial. Nat. Med..

[38] Sacher A., LoRusso P., Patel M.R., Miller W.H., Garralda E., Forster M.D., Santoro A., Falcon A., Kim T.W., Paz-Ares L. (2023). Single-Agent Divarasib (GDC-6036) in Solid Tumors with a KRAS G12C Mutation. N. Engl. J. Med..

[39] Shi Y., Fang J., Xing L., Yao Y., Zhang J., Liu L., Wang Y., Hu C., Xiong J., Liu Z. (2025). Glecirasib in *KRAS*^G12C^-mutated nonsmall-cell lung cancer: A phase 2b trial. Nat. Med..

[40] Li J., Wang Z., Huang J., Ba Y., Cao B., Luo S., Li W., Bai C., Song Z., Xiong J. (2026). Glecirasib with or without cetuximab in previously treated locally advanced or metastatic colorectal cancer with *KRAS*^G12C^ mutation (JAB-21822-1002 and JAB-21822-1007): Two open-label, non-randomised phase 1/2 trials. Lancet Gastroenterol. Hepatol..

[41] Peng S., Zhang Y., Lin X., Si C., Van Horn R.D., Dempsey J.A., Goetz E., Evans R.J., Farber A., Vandekopple M.J. (2026). Characterization of KRAS^G12C^ inhibitor olomorasib single-agent and combination with activity in KRAS^G12C^-mutant models. Nat. Commun..

[42] Murciano-Goroff Y.R., Hollebecque A., Heist R.S., Cassier P.A., Han J.Y., Kim S.Y., Sabari J.K., Tosi D., Sacher A., Burns T.F. (2026). Pan-tumor activity of olomorasib, a next-generation KRAS G12C inhibitor in *KRAS* G12C-mutant advanced solid tumors: A first-in-human study. Nat. Commun..

[43] Ruan D.Y., Wu H.X., Xu Y., Munster P.N., Deng Y., Richardson G., Yan D., Lee M.A., Lee K.W., Pan H. (2025). Garsorasib, a KRAS G12C inhibitor, with or without cetuximab, an EGFR antibody, in colorectal cancer cohorts of a phase II trial in advanced solid tumors with KRAS G12C mutation. Signal Transduct. Target. Ther..

[44] Yamamoto N., Yan D., Ganju V., Hou X., Pan H., Shan J., Wang L., Kim S.W., Richardson G., Sanborn R.E. (2026). Efficacy and safety of garsorasib in patients with KRAS G12C-mutated advanced pancreatic cancer. Br. J. Cancer.

[45] Lorthiois E., Gerspacher M., Beyer K.S., Vaupel A., Leblanc C., Stringer R., Weiss A., Wilcken R., Guthy D.A., Lingel A. (2022). JDQ443, a Structurally Novel, Pyrazole-Based, Covalent Inhibitor of KRAS^G12C^ for the Treatment of Solid Tumors. J. Med. Chem..

[46] Cappuzzo F., Castro G., Kang J.H., Wu Y.L., Brustugun O.T., Cheema P.K., Owonikoko T.K., Longin A.S., Duan J., Caparica R. (2024). KontRASt-02: A Phase III Trial Investigating the Efficacy and Safety of the KRAS^G12C^ Inhibitor JDQ443 vs. Docetaxel in Patients with Previously Treated, Locally Advanced or Metastatic, KRAS G12C-Mutated NSCLC. Int. J. Radiat. Oncol. Biol. Phys..

[47] Ernst S.M., van Marion R., Atmodimedjo P.N., de Jonge E., Mathijssen R.H.J., Paats M.S., de Bruijn P., Koolen S.L., von der Thüsen J.H., Aerts J. (2024). Clinical Utility of Circulating Tumor DNA in Patients With Advanced *KRAS*^G12C^-Mutated NSCLC Treated With Sotorasib. J. Thorac. Oncol..

[48] Morgillo F., Della Corte C.M., Fasano M., Ciardiello F. (2016). Mechanisms of resistance to EGFR-targeted drugs: Lung cancer. ESMO Open.

[49] Yoda S., Lin J.J., Lawrence M.S., Burke B.J., Friboulet L., Langenbucher A., Dardaei L., Prutisto-Chang K., Dagogo-Jack I., Timofeevski S. (2018). Sequential ALK Inhibitors Can Select for Lorlatinib-Resistant Compound ALK Mutations in ALK-Positive Lung Cancer. Cancer Discov..

[50] Valencia-Sama I., Ladumor Y., Kee L., Adderley T., Christopher G., Robinson C.M., Kano Y., Ohh M., Irwin M.S. (2020). NRAS Status Determines Sensitivity to SHP2 Inhibitor Combination Therapies Targeting the RAS-MAPK Pathway in Neuroblastoma. Cancer Res..

[51] Kemp S.B., Cheng N., Markosyan N., Sor R., Kim I.K., Hallin J., Shoush J., Quinones L., Brown N.V., Bassett J.B. (2023). Efficacy of a Small-Molecule Inhibitor of Kras^G12D^ in Immunocompetent Models of Pancreatic Cancer. Cancer Discov..

[52] Romero J.M., Grünwald B., Jang G.H., Bavi P.P., Jhaveri A., Masoomian M., Fischer S.E., Zhang A., Denroche R.E., Lungu I.M. (2020). A Four-Chemokine Signature Is Associated with a T-cell-Inflamed Phenotype in Primary and Metastatic Pancreatic Cancer. Clin. Cancer Res..

[53] Issahaku A.R., Mukelabai N., Agoni C., Rudrapal M., Aldosari S.M., Almalki S.G., Khan J. (2022). Characterization of the binding of MRTX1133 as an avenue for the discovery of potential KRAS^G12D^ inhibitors for cancer therapy. Sci. Rep..

[54] ClinicalTrials.gov (2025). Study of MRTX1133 in Patients with Advanced Solid Tumors Harboring a KRAS G12D Mutation. https://clinicaltrials.gov/study/NCT05737706.

[55] ClinicalTrials.gov Study of RMC-9805 in Participants with KRAS G12D-Mutant Solid Tumors 2024. https://clinicaltrials.gov/study/NCT06040541.

[56] Yaeger R., Chatila W.K., Lipsyc M.D., Hechtman J.F., Cercek A., Sanchez-Vega F., Jayakumaran G., Middha S., Zehir A., Donoghue M.T.A. (2018). Clinical Sequencing Defines the Genomic Landscape of Metastatic Colorectal Cancer. Cancer Cell.

[57] Kim D., Herdeis L., Rudolph D., Zhao Y., Böttcher J., Vides A., Ayala-Santos C.I., Pourfarjam Y., Cuevas-Navarro A., Xue J.Y. (2023). Pan-KRAS inhibitor disables oncogenic signalling and tumour growth. Nature.

[58] Wolpin B.M., Park W., Garrido-Laguna I., Spira A., Starodub A., Sommerhalder D., Punekar S.R., Barve M., Pelster M., Herzberg B. (2026). Daraxonrasib in Previously Treated Advanced RAS-Mutated Pancreatic Cancer. N. Engl. J. Med..

[59] O’Reilly E.M., Wainberg Z.A., Hendifar A.E., Borad M.J., Pietrantonio F., Pant S., Hammel P., Cremolini C., Manji G.A., Oberstein P.E. (2026). Daraxonrasib or Chemotherapy in Previously Treated Metastatic Pancreatic Cancer. N. Engl. J. Med..

[60] Hofmann M.H., Gmachl M., Ramharter J., Savarese F., Gerlach D., Marszalek J.R., Sanderson M.P., Kessler D., Trapani F., Arnhof H. (2021). BI-3406, a Potent and Selective SOS1-KRAS Interaction Inhibitor, Is Effective in KRAS-Driven Cancers through Combined MEK Inhibition. Cancer Discov..

[61] Bond M.J., Chu L., Nalawansha D.A., Li K., Crews C.M. (2020). Targeted Degradation of Oncogenic KRAS^G12C^ by VHL-Recruiting PROTACs. ACS Cent. Sci..

[62] Popow J., Farnaby W., Gollner A., Kofink C., Fischer G., Wurm M., Zollman D., Wijaya A., Mischerikow N., Hasenoehrl C. (2024). Targeting cancer with small-molecule pan-KRAS degraders. Science.

[63] Zhou C., Fan Z., Gu Y., Ge Z., Tao Z., Cui R., Li Y., Zhou G., Huo R., Gao M. (2024). Design, Synthesis, and Biological Evaluation of Potent and Selective PROTAC Degraders of Oncogenic KRAS^G12D^. J. Med. Chem..

[64] Kos T., Saur D. (2025). Breaking down KRAS: Small-molecule degraders for cancer therapy. Signal Transduct. Target. Ther..

[65] Wang D., Wang Q., Wang Y., Chen P., Lu X., Jia F., Sun Y., Sun T., Zhang L., Che F. (2022). Targeting oncogenic KRAS with molecular brush-conjugated antisense oligonucleotides. Proc. Natl. Acad. Sci. USA.

[66] Kalluri V.S., Smaglo B.G., Mahadevan K.K., Kirtley M.L., McAndrews K.M., Mendt M., Yang S., Maldonado A.S., Sugimoto H., Salvatierra M.E. (2025). Engineered exosomes with Kras^G12D^ specific siRNA in pancreatic cancer: A phase I study with immunological correlates. Nat. Commun..

[67] Pant S., Wainberg Z.A., Weekes C.D., Furqan M., Kasi P.M., Devoe C.E., Leal A.D., Chung V., Basturk O., VanWyk H. (2024). Lymph-node-targeted, mKRAS-specific amphiphile vaccine in pancreatic and colorectal cancer: The phase 1 AMPLIFY-201 trial. Nat. Med..

[68] Linette G.P., Bear A.S., Carreno B.M. (2024). Facts and Hopes in Immunotherapy Strategies Targeting Antigens Derived from KRAS Mutations. Clin. Cancer Res..

[69] Cafri G., Gartner J.J., Zaks T., Hopson K., Levin N., Paria B.C., Parkhurst M.R., Yossef R., Lowery F.J., Jafferji M.S. (2020). mRNA vaccine-induced neoantigen-specific T cell immunity in patients with gastrointestinal cancer. J. Clin. Investig..

[70] Lu D., Chen Y., Jiang M., Wang J., Li Y., Ma K., Sun W., Zheng X., Qi J., Jin W. (2023). KRAS G12V neoantigen specific T cell receptor for adoptive T cell therapy against tumors. Nat. Commun..

[71] Bery N., Legg S., Debreczeni J., Breed J., Embrey K., Stubbs C., Kolasinska-Zwierz P., Barrett N., Marwood R., Watson J. (2019). KRAS-specific inhibition using a DARPin binding to a site in the allosteric lobe. Nat. Commun..

[72] Zhang Z., Gao R., Hu Q., Peacock H., Peacock D.M., Dai S., Shokat K.M., Suga H. (2020). GTP-State-Selective Cyclic Peptide Ligands of K-Ras(G12D) Block Its Interaction with Raf. ACS Cent. Sci..

[73] Kauke M.J., Traxlmayr M.W., Parker J.A., Kiefer J.D., Knihtila R., McGee J., Verdine G., Mattos C., Wittrup K.D. (2017). An engineered protein antagonist of K-Ras/B-Raf interaction. Sci. Rep..

[74] Ranđelović I., Nyíri K., Koppány G., Baranyi M., Tóvári J., Kigyós A., Tímár J., Vértessy B.G., Grolmusz V. (2024). Gluing GAP to RAS Mutants: A New Approach to an Old Problem in Cancer Drug Development. Int. J. Mol. Sci..

[75] Arbour K.C., Jordan E., Kim H.R., Dienstag J., Yu H.A., Sanchez-Vega F., Lito P., Berger M., Solit D.B., Hellmann M. (2018). Effects of Co-occurring Genomic Alterations on Outcomes in Patients with KRAS-Mutant Non-Small Cell Lung Cancer. Clin. Cancer Res..

[76] Skoulidis F., Goldberg M.E., Greenawalt D.M., Hellmann M.D., Awad M.M., Gainor J.F., Schrock A.B., Hartmaier R.J., Trabucco S.E., Gay L. (2018). STK11/LKB1 Mutations and PD-1 Inhibitor Resistance in KRAS-Mutant Lung Adenocarcinoma. Cancer Discov..

[77] Kotani D., Oki E., Nakamura Y., Yukami H., Mishima S., Bando H., Shirasu H., Yamazaki K., Watanabe J., Kotaka M. (2023). Molecular residual disease and efficacy of adjuvant chemotherapy in patients with colorectal cancer. Nat. Med..

[78] Tie J., Cohen J.D., Lahouel K., Lo S.N., Wang Y., Kosmider S., Wong R., Shapiro J., Lee M., Harris S. (2022). Circulating Tumor DNA Analysis Guiding Adjuvant Therapy in Stage II Colon Cancer. N. Engl. J. Med..

[79] Parikh A.R., Leshchiner I., Elagina L., Goyal L., Levovitz C., Siravegna G., Livitz D., Rhrissorrakrai K., Martin E.E., Van Seventer E.E. (2019). Liquid versus tissue biopsy for detecting acquired resistance and tumor heterogeneity in gastrointestinal cancers. Nat. Med..

[80] Noji R., Tohyama K., Nakamura S., Naito T., Oikawa Y., Kuroshima T., Tomioka H., Michi Y., Ikeda S., Asakage T. (2024). Dynamic Changes in Circulating Tumor DNA During Immunotherapy for Head and Neck Cancer: SHIZUKU-HN Study. Int. J. Mol. Sci..

[81] Wan J.C.M., Massie C., Garcia-Corbacho J., Mouliere F., Brenton J.D., Caldas C., Pacey S., Baird R., Rosenfeld N. (2017). Liquid biopsies come of age: Towards implementation of circulating tumour DNA. Nat. Rev. Cancer.

